# Review of "Gene Regulation and Metabolism" edited by Julio Collado-Vides and Ralf Hofestädt

**DOI:** 10.1186/1475-925X-4-47

**Published:** 2005-08-08

**Authors:** Francisco Azuaje

**Affiliations:** 1 Computer Science Research Institute, University of Ulster, Jordanstown, Co. Antrim, BT37 0QB, Northern Ireland, UK

The inference, analysis and integration of networks of genes and products are fundamental goals of post-genomic research. Over the past few years several data- and knowledge-driven computational approaches have been proposed, which have provided relevant insights into the informational complexity and function of some of such networks in different organisms. *Gene Regulation and Metabolism *probably represents one of the first books attempting to discuss essential computational concepts, methods and applications in this area. Although its editors did not aim to offer a comprehensive review of advances available at the time of its publication, this book may still be seen as a useful introduction for computer scientists to key problems, solutions and questions relevant to the study of regulatory and metabolic networks.

Collado-Vides and Hofestädt edited a collection of contributions by internationally-recognised authors, which were motivated by a summer school following a series of Dagstuhl seminars in Germany. The book is divided into three main parts addressing information and knowledge representation problems, gene regulation and post-genomic approaches. Each chapter also provides lists of suggested readings and relevant websites, which include some of the databases and tools discussed in the book. The book also offers a glossary of terms and acronyms. Overall, the chapters are well-written and organised. Readers would have benefited from an introductory chapter to overview fundamental biological concepts and problems. However, this is partially compensated by some chapters, which adequately present basic concepts and motivation necessary to approach specific applications such as DNA regulatory motif discovery.

In the first chapter, Ahouse discusses the concept of homology and its inference within the context of regulatory networks, which represents an important source for predicting potential interactions. The second chapter, by Apweiler *et al.*, describes several techniques for automated protein sequence characterisation and its applications in whole proteome analysis. Chapter 3, by Freier *et al.*, overviews approaches to database integration, which are relevant to the prediction and analysis of metabolic networks. They also put emphasis on the application of a rule-based simulation tool (*MetabSim*). In Chapter 4, Stormo introduces quantitative indicators required to support the prediction of regulatory binding sites from sets of co-regulated genes. This is followed by Collado-Vides *et al.*'s review of methods for identifying regulatory elements, e.g. promoters and regulatory binding sites. Chapter 6, by Manson McGuire and Church, focuses on the discovery of DNA regulatory motifs. It introduces different techniques for regulon prediction and an algorithm for detecting motifs (*AlignACE*). In Chapter 7, Kolchanov *et al. *discusses the representation and modelling of gene networks using the *GeneNet *database. This system encodes different types of networks, e.g. networks relating to cell growth and differentiation. This chapter concludes with an introduction to the mathematical simulation of gene network dynamics. Chapter 8, by Huang, emphasises Boolean networks approaches to modelling cellular regulatory systems. In Chapter 9, Marcotte discusses important questions and challenges for the large-scale prediction of protein function and networks. Chapter 10, by Schmidt and Dandekar, concentrates on metabolic pathways. It introduces fundamental concepts and problems, such as enzymatic reactions and pathway alignment. The final chapter, by Tomita, introduces the *E-CELL *project, which has been applied to simulate several cellular processes, e.g. signal transduction and gene regulation. The ultimate goal of this project is to model the cell based on large-scale representations of thousands of functionally-diverse processes.

Even when it does not provide a comprehensive, up-to-date review of techniques and tools for supporting the inference and analysis of gene regulation and metabolism networks, this book represents a useful reference to reflect on recent advances and challenges ahead. Moreover, some of its chapters highlight crucial open questions and computational opportunities in the systems biology era.

